# Comparison of safety and efficacy in venous access closure using a double purse string suture technique vs. Z-suture technique after MitraClip procedure

**DOI:** 10.3389/fcvm.2025.1585522

**Published:** 2025-07-25

**Authors:** Anas Alnaimi, Sebastian Frederik Mause, Nawar Alachkar, Jörg W. Schröder, Mathias Burgmaier, Ertunc Altiok, Nikolaus Marx, Sebastian Reith, Mohammad Almalla

**Affiliations:** ^1^ Department of Internal Medicine I, Cardiology, University Hospital Aachen, RWTH Aachen University, Aachen, Germany; ^2^ Faculty of Applied Healthcare Science, Deggendorf Institute of Technology, Deggendorf, Germany; ^3^ Department of Internal Medicine III, St. Franziskus Hospital, Münster, Germany; ^4^ Madinah Cardiac Center, Medina, Saudi Arabia

**Keywords:** MitraClip, percutaneous mitral valve repair, vascular complications, venous access closure, double purse string suture, Z-suture

## Abstract

**Background:**

Percutaneous mitral valve repair using the MitraClip System is a well-established therapeutic option for patients with symptomatic mitral regurgitation. This procedure is usually performed via venous femoral access using a 24-French guiding catheter. Since vascular complications and bleeding remain a relevant limitation, we now compared access closure using subcutaneous absorbable double purse string suture (DPSS) and Z-suture technique following MitraClip procedure.

**Methods:**

249 patients (mean age 76 ± 8 years) who underwent MitraClip procedure at our institution were included. Venous closure was performed using Z-suture technique in 140 patients and DPSS technique in 109 patients. Vascular complications and bleeding events were assessed according to the Mitral Valve Academic Research Consortium (MVARC) criteria.

**Results:**

MVARC minor and major vascular complications were comparable after closure with Z-suture and DPSS-technique (4.3% vs. 0.9%, *p* = 0.11 and 1.4% vs. 0.9%, *p* = 0.71, respectively). However, development of AV-fistula and requirement of access related surgical intervention was more often observed in the Z-suture group (5% vs. 0%, *p* = 0.018 and 3.5% vs. 0%, *p* = 0.045). MVARC minor and major, non-life-threatening bleeding did not differ between the two groups (10.7% vs. 12.9%, *p* = 0.61 and 0.7% vs. 0.0%, *p* = 0.38). Similarly, overall transfusion rates and access related blood transfusion rates were comparable (11.4% vs. 15.5%, *p* = 0.34 and 4.3% vs. 2.7%, *p* = 0.52).

**Conclusion:**

Large caliber venous access closure with DPSS technique was feasible, safe, and effective to achieve haemostasis after MitraClip procedure. Compared with Z-suture, use of DPSS closure was associated with a lower rate of required access related surgical intervention and postinterventional AV-fistula.

## Introduction

Mitral regurgitation is common among patients with heart failure and is associated with a poor prognosis (1, 2). Among patients with heart failure and moderate to severe functional mitral regurgitation who receive medical therapy, the addition of Percutaneous mitral valve repair (PMVR) (also known as transcatheter mitral-valve repair) is an effective and safe treatment option, potentially reducing the risk of hospitalization for heart failure or cardiovascular death (2–4). Large caliber venous access with 24-French through femoral vein is required to deliver MitraClip device (Abbott Vascular, Santa Clara, CA). Notably, vascular and bleeding complications have been shown to the most frequent major complications of PMVR and to be associated with adverse outcomes and subsequent mortality in patients undergoing surgical or transcatheter valve procedures (5–7). Generally, patients with relevant mitral valve disease undergoing PMVR are typically prone to vascular injury and bleeding due to their abundant and multifaceted underlying comorbidities, frailty, and frequent use of anticoagulation. Consequently, optimization of access site management represents a keystone in addressing procedure-related adverse events.

Closure of the venous access site after MitraClip is usually achieved by manual compression, subcutaneous suture closure techniques or application of a closure system, however a standardization is so far lacking (8–11). To date, only Perclose ProGlide suture (Abbott Vascular, Santa Clara, CA) mediated closure system is approved to achieve haemostasis after removal of venous access sheaths up to 24-French. Over the last decade, several methods were evaluated to achieve immediate haemostasis after removal of larger guiding catheter. Some studies evaluated the feasibility and safety of Z-suture technique (also known as figure-of-eight suture) for venous access closure (10–13). Percutaneous skin closure using double purse string suture (DPSS) was also evaluated to achieve immediate haemostasis after using large caliber venous access, e.g., following leadless pacemaker implantation through femoral vein access with sheath sizes up to 23-French (14). To date, there is no data regarding the application of the subcutaneous absorbable DPSS technique after MitraClip procedure. Considering the need for optimization of access site management, we now compared in a monocentric all-comer study the feasibility, efficacy, and safety of the Z-suture and DPSS technique for venous femoral access-closure after PMVR using the MitraClip.

## Methods

### Study design and patient population

This a single centre, non-randomized all-comer study that included 249 patients with symptomatic, moderate-to-severe or severe mitral regurgitation and high surgical risk undergoing PMVR using the MitraClip. All consecutive patients who underwent PMVR at the Heart Centre of the University Hospital of Aachen, Germany, between January 2014 and December 2017 were eligible for inclusion in our registry. Closure of femoral vein access was performed with Z-suture technique between January 2014 and December 2015 (140 patients) and the subcutaneous absorbable DPSS technique between January 2016 and December 2017 (109 patients) for achieving vascular haemostasis after sheath removal. All patients had an indication for treatment of mitral regurgitation according to current guidelines at the time (15, 16), and were discussed with cardiac surgeons in a multidisciplinary heart team. Only patients at high or prohibitive surgical risk and suitable to PMVR using the MitraClip were treated. The involved operators had at least five years of experience in large caliber venous access management before initiation of the study. All research was performed in accordance with the relevant regulations and informed consent was exempted by the ethics committee of the Faculty of Medicine, University RWTH Aachen, Germany.

### Endpoints

Unless stated otherwise, efficacy and safety endpoints were assessed and defined according to the Mitral Valve Academic Research Consortium (MVARC) criteria with a modified Valve Academic Research Consortium (VARC 2) classification scheme (6). In addition to the analysis of vascular complications and access site related bleeding following transcatheter valve repair, we assessed the need for vascular surgery and endovascular intervention as well as major adverse cardiac and cerebrovascular events (MACCE) and in-hospital mortality.

### MitraClip procedure and peri-interventional management

Following puncture of the right femoral vein as the standard vascular access and echocardiographic-guided transseptal puncture, a heparin bolus of 100 U/kg was given to achieve an activated clotting time (ACT) of 300–350 s ACT was measured at 15 min intervals and an additional bolus of heparin was administered when necessary to maintain an ACT of 300–350 s A 24-French guiding catheter was advanced from the right atrium to the left atrium. The MitraClip device was deployed under guidance of real-time 3-dimensional transoesophageal echocardiography. All procedure was performed under general anaesthesia.

### Suture technique

#### Z-suture technique

**Figure 1:**
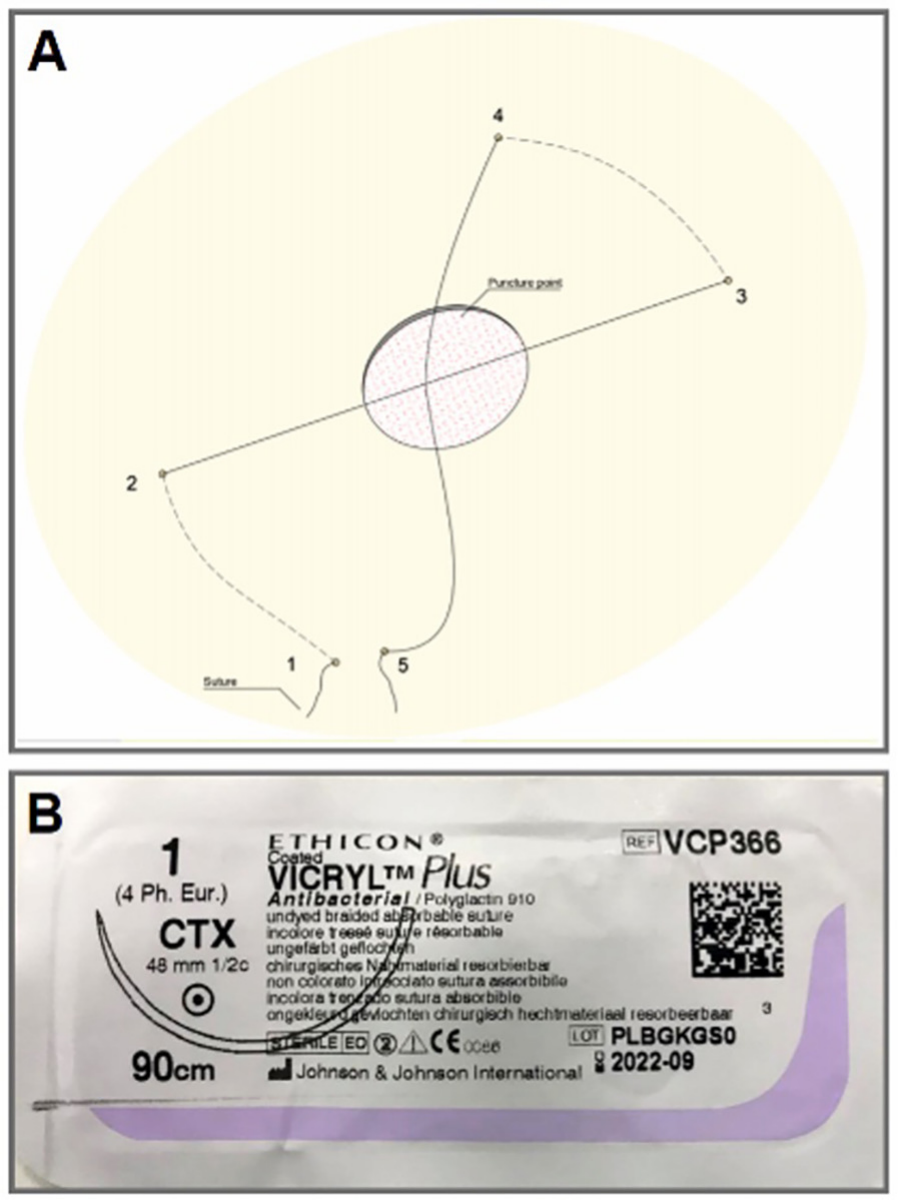
Z-suture technique. Panel **(A)**: synopsis of the basic steps of Z-suture. Panel **(B)**: 48 mm, ½ circular needle attached to 90 cm silk.

A silk suture attached to a large cutting curved needle (48 mm,1/2 C) was passed through subcutaneous tissue distal to the sheath entry site from medial to lateral, superficial to the level of femoral vessels. Subsequently, the needle and suture were passed again, using the same technique proximal to the entry site of the sheath from medial to lateral. After sheath removal, the suture was tightened, resulting in shape of “Z” to deploy enough pressure for effective haemostasis (Figure 1). If needed, additional knots were added to reinforce the closure. Furthermore, a circular compression bandage was applied. The compression bandage and suture were removed after 8 h.

#### Subcutaneous absorbable DPSS technique

**Figure 2:**
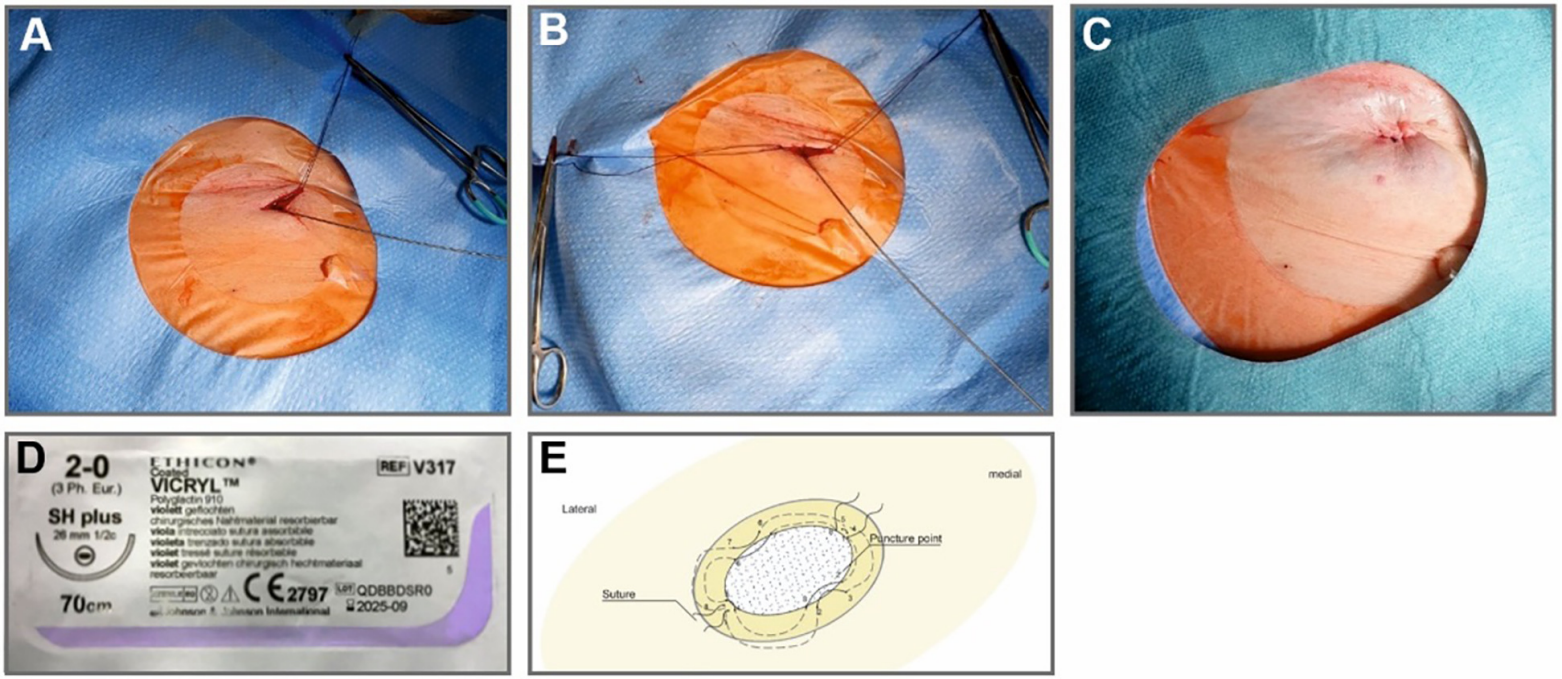
Subcutaneous absorbable double purse string suture (DPSS). Panel **(A)**: First subcutaneous absorbable suture in place. Panel **(B)**: Both sutures in place. Panel **(C)**: Closure of venous access after removal of 24-French sheath. Panel **(D)**: 26 mm, ½ circular needle attached to 70 cm absorbable silk. Panel **(E)**: synopsis of the basic steps of DPPS.

After puncture of the femoral vein, a 1 cm-incision was made in the puncture site. Subsequently, a continuous running suture was performed around the opening incision using absorbable silk suture attached to small cutting curved needle (26 mm, 1/2C). The suture was performed twice, first from lateral to medial and then from medial to lateral. After completion of the procedure and sheath removal, both sutures were drawn tightly (Figure 2). Furthermore, a circular compression bandage was applied. The compression bandage was removed after for 8 h.

Heparin reversal with protamine after the MitraClip procedure was not routinely administrated in both groups.

### Post-interventional management and follow up after MitraClip procedure

All patients were transported from catheter lab to our intermediate or intensive care unit for overnight monitoring, and then transferred to standard care ward for 3–5 days. Physical examination was performed directly after removal of the bandage and on the next day to detect any vascular complication including hematoma, iatrogenic pseudoaneurysm or iatrogenic arteriovenous (AV)—fistula. If a vascular complication was suspected, patients were sent immediately to our angiology unit to perform Duplex-Doppler sonography. Independently, all patients received routinely a Duplex-Doppler sonography of the access site before discharge. In patients with AV-fistula, shunt volume was calculated as following: diameter of the femoral artery and velocity time integral of the femoral flow using PW Doppler was measured proximal to the iatrogenic AV fistula at both sides. The flow volume through both left and right femoral artery were calculated using the following equation: Flow = 2π (femoral artery diameter/2)2*VTI in ml/minute. The difference of flow volumes between both sides indicated the shunt volume. A significant shunt was defined as a shunt volume of more than 1,000 ml/min, presence of high diastolic pressure in femoral artery segment proximal to the AV-fistula or presence of monophasic flow signal in segment of femoral artery distal to the AV-fistula.

### Statistical analysis

Patients were classified in two groups: Z-suture group and DPSS group. Baseline clinical and echocardiographic characteristics of patients were assessed. Continuous variables were expressed as mean ± standard deviation and binary variables were expressed as count (percentage). Differences in the 2 groups were evaluated using Fisher's exact tests for categorical variables and unpaired *t* tests or Mann–Whitney *U* tests for continuous variables, depending on normality. Statistical analyses were performed with SPSS version 25.0 (IBM Corp., Armonk, NY, USA). Statistical significance was awarded by *p* < 0.05.

## Results

### Baseline characteristics

A total of 249 patients were enrolled during January 2014 and December 2017. Baseline demographic, clinical and echocardiographic characteristics are shown in Tables 1, 2. Patients were elderly (mean age 75 ± 8 years), dominantly male (66%) and most had advanced symptoms (New York Heart Association functional class III or IV in 79.1%). Relevant morbidities, such as coronary artery disease, atrial fibrillation, chronic kidney disease, prior stroke, diabetes mellitus, chronic obstructive pulmonary disease and prior sternotomy were common in our single-centre cohort and patients were at high surgical risk (logistic EuroSCORE 24.7 ± 16%). The predominant pathology treated with transcatheter mitral valve repair was functional mitral regurgitation (MR; 68.9%) and the MitraClip procedure was performed for grade III or IV MR in 84.7%. Preprocedural echocardiography revealed a mean ventricular ejection fraction of 38.3 ± 13%, a mean effective regurgitant orifice area (EROA) of 34.4 ± 14 mm^2^ and a mean regurgitant volume (RVol) of 54.9 ± 20 ml. The procedure was successfully completed with implantation of at least one clip and reduction of MR to maximally moderate MR in 95.3% of patients.

**Table 1:** Baseline characteristics of all patients.

Variable	Z-suture *N* = 140	DPSS *N* = 109	*p*-value
Age, years	75 ± 9	76 ± 7	0.118
Male, *n* (%)	93 (66%)	72 (66%)	0.950
Hypertension, *n* (%)	102 (73%)	74 (68%)	0.393
Body mass index, kg/m²	26.2 ± 4.7	26.0 ± 5.2	0.695
Diabetes mellitus, *n* (%)	45 (32%)	33 (30%)	0.753
Chronic kidney disease, *n* (%)	102 (73%)	84 (77%)	0.449
End-stage renal disease, *n* (%)	8 (6%)	6 (5%)	0.943
Coronary artery disease, *n* (%)	105 (75%)	77 (71%)	0.442
Dilatative cardiomyopathy, *n* (%)	35 (25%)	22 (20%)	0.370
Previous MI, *n* (%)	48 (34%)	36 (33%)	0.835
Previous CABG, *n* (%)	41 (29%)	20 (18%)	**0** **.** **046**
Previous Stroke, *n* (%)	19 (14%)	8 (7%)	0.117
COPD, *n* (%)	64 (46%)	41 (38%)	0.510
Atrial fibrillation, *n* (%)	99 (70%)	78 (72%)	0.884
Logistic EuroSCORE (%)	26 ± 12	23 ± 19	0.056
Pacemaker/ ICD, *n* (%)	44 (31%)	26 (24%)	0.188
CRT, *n* (%)	15 (11%)	8 (7%)	0.362
NYHA class, *n* (%)			
II	36 (26%)	14 (13%)	**0** **.** **012**
III	63 (45%)	75 (69%)	**<0** **.** **001**
IV	41 (29%)	20 (18%)	0.113

Bold values denote statistical significance at the *p* < 0.05 level.

BMI, body mass index; CABG, coronary artery bypass grafting; COPD, chronic obstructive pulmonary disease; CRT, cardiac resynchronization therapy; ICD, implantable cardioverter defibrillator; MI, myocardial infarction; NYHA, New York heart failure association.

**Table 2:** Echocardiographic and hemodynamic characteristics of patients.

Variable	Z-suture, *N* = 140 (%)	DPSS, *N* = 109 (%)	*p*-value
Echocardiographic parameters			
Mitral regurgitation grade, *n* (%)			
≥III	126 (90%)	85 (80%)	**0** **.** **009**
<III	14 (10%)	24 (20%)	**0** **.** **009**
PISA, mm	8.8 ± 2.2	8.9 ± 2.4	0.620
EROA, mm²	37 ± 18	31 ± 11	**0** **.** **019**
Regurgitant volume, ml/beat	58 ± 28	51 ± 16	**0** **.** **040**
Etiology of MR, *n* (%)			
Primary	43 (31%)	35 (32%)	0.452
Functional	97 (69%)	74 (68%)	0.452
Left ventricular ejection fraction, %	37 ± 13	40 ± 14	0.116
Mean pressure gradient after MitraClip, mmHg	3.7 ± 1.8	3.9 ± 1.6	0.369
Reduction of MR grade after MitraClip	2.4 ± 0.7	2.2 ± 1.0	0.208
Number of clips per patients, n	1.4 ± 0.6	1.7 ± 0.8	**0** **.** **0007**
Tricuspid regurgitation grade, *n* (%)			
I-II	82 (59%)	72 (66%)	0.228
>II	58 (41%)	37 (34%)	0.228

Bold values denote statistical significance at the *p* < 0.05 level.

EROA, effective regurgitation orifice area; MR, mitral regurgitation; PISA, proximal isovelocity surface area.

There were no significant differences concerning demographic parameters, especially parameters that may be affected access site closure efficacy and safety between both groups [age, sex, body mass index (BMI), chronic kidney disease and anticoagulant therapy]. MR grade ≥ III was more often in Z-suture group compared to DPSS group (90 vs. 80%, *p* = 0.009, respectively). Correspondingly, EROA and RVol was greater in the Z-suture group (37 vs. 31 mm^2^, *p* = 0.019; 58 vs. 51 ml, *p* = 0.040, respectively). More than one clip was implanted in less patients in the Z-suture group compared to the DPSS group (1.4 ± 0.6 vs. 1.7 ± 0.8; respectively, *p* < 0.001). The postprocedural mean transmitral pressure gradient (3.7 ± 1.8 vs. 3.9 ± 1.6 mmHg; respectively, *p* = 0.37) and the degree of mitral regurgitation grade reduction following PMVR (2.4 ± 0.7 vs. 2.2 ± 1.0, *p* = 0.21) did not significantly differ among both groups.

### Vascular complications and bleeding

Closure of femoral vein access was performed using the Z-suture technique in 140 patients, whereas DPSS technique was used in the subsequent 109 patients. Incidence of vascular complications were in line with previous reports of real-world data following PMVR (5, 7, 17). Access related MVARC minor vascular complications occurred in six patients from the Z-suture group and in one patient from the DPSS group (4.3% vs. 0.9%, *p* = 0.111, respectively). MVARC major vascular complications were observed in two patients from the Z-suture group and one patient from the DPSS group (1.4% vs. 0.9%, *p* = 0.714, respectively). Table 3 summarizes access site vascular complications.

**Table 3:** In-hospital clinical outcome.

Outcome	Z-suture, *N* = 140	DPSS, *N* = 109	*p*-value
Vascular complications			
MVARC vascular minor complication, *n* (%)	6 (4.3%)	1 (0.9%)	0.111
MVARC vascular major complication, *n* (%)	2 (1.4%)	1 (0.9%)	0.714
Vascular obstruction, *n* (%)	0 (0%)	0 (0%)	1.000
Vascular dissection, *n* (%)	1 (0.7%)	0 (0%)	0.377
Vascular perforation, *n* (%)	0 (0%)	1 (0.9%)	0.257
AV fistula, *n* (%)	7 (5%)	0 (0%)	**0** **.** **018**
Pseudoaneurysms, *n* (%)	4 (2.8%)	0 (0%)	0.075
Superficial Hematoma, *n* (%)	16 (11%)	13 (11.9%)	0.903
Small < 5 cm	8 (5.7%)	3 (2.7%)	0.260
Large > 5 cm	8 (5.7%)	10 (9.1%)	0.268
Access related bleeding and others			
MVARC minor bleeding, *n* (%)	15 (10.7%)	14 (12.9%)	0.604
MVARC major, non-life-threatening bleeding, *n* (%)	1 (0.7%)	0 (0%)	0.377
Life threatening/disabling bleeding, *n* (%)	1 (0.7%)	1 (0.9%)	0.858
Transfusion, *n* (%)	16 (11.4%)	17 (15.5%)	0.336
Access related blood transfusions, *n* (%)	6 (4.3%)	3 (2.7%)	0.521
Vascular surgery, *n* (%)	5 (3.5%)	0 (0%)	**0** **.** **046**
Endovascular intervention, *n* (%)	2 (1.4%)	1 (0.9%)	0.714
Access site infection, *n* (%)	3 (2.1%)	0 (0%)	0.124
Further adverse clinical events			
In-hospital mortality, *n* (%)	2 (1.4%)	3 (2.7%)	0.461
In-hospital MACCE, *n* (%)	5 (3.5%)	5 (4.5%)	0.686

Bold values denote statistical significance at the *p* < 0.05 level.

AV, arteriovenous; MVARC, mitral valve academic research consortium; MACCE, major adverse cardiac and cerebrovascular events.

### Differentiation of vascular complications

#### Iatrogenic arteriovenous (AV) fistula

Postprocedural AV fistula was observed in seven of 249 patients (2.8%), whereas all these patients were in the Z-suture group (5% vs. 0%, *p* = 0.018). A significant shunt volume as assessed by Duplex-Doppler sonography (1,950 ± 450 ml) was observed in six patients. One patient had a non-significant shunt volume (300 ml) and one patient had additionally a major bleeding.

#### Iatrogenic pseudoaneurysm

A total of four patients developed an iatrogenic pseudoaneurysm. All these patients were in Z-suture group, whereas no pseudoaneurysm was observed in patients in the DPSS group (2.8% vs. 0%, respectively, *p* = 0.075). One patient was treated surgically, and three patients were treated with ultrasound guided thrombin injection.

#### Other vascular complications

Vascular obstruction did not occur in any patient. Vascular dissection occurred only in one patient from the Z-suture groups, whereas vascular perforation occurred only in one patient from the DPSS group. There was no difference in the incidence of superficial hematoma between both groups; 16 patients in Z-suture group and 13 patients in the DPSS group (11.0% vs. 11.9%, *p* = 0.903, respectively). Three patients in the Z-suture group had access site related infections due to delayed removal of Z-suture.

### Access site related bleeding

Minor bleeding according to the MVARC criteria occurred in 15 patients (10.7%) from the Z-suture group compared to 14 patients (12.9%) from the DPSS group (*p* = 0.604). Major, non- lifethreatening bleeding occurred only in one patient in the Z-suture group (0.7%) and in no patient in the DPSS group (*p* = 0.377). Life threatening bleeding occurred in one patient from both groups (*p* = 0.855).

Furthermore, no significant difference between both groups was detected in the total blood transfusion rate (11.4% vs. 15.5%, *p* = 0.336) as well as the access related blood transfusion rate (4.3% vs. 2.7%, *p* = 0.521).

### Vascular complications requiring intervention

**Figure 3:**
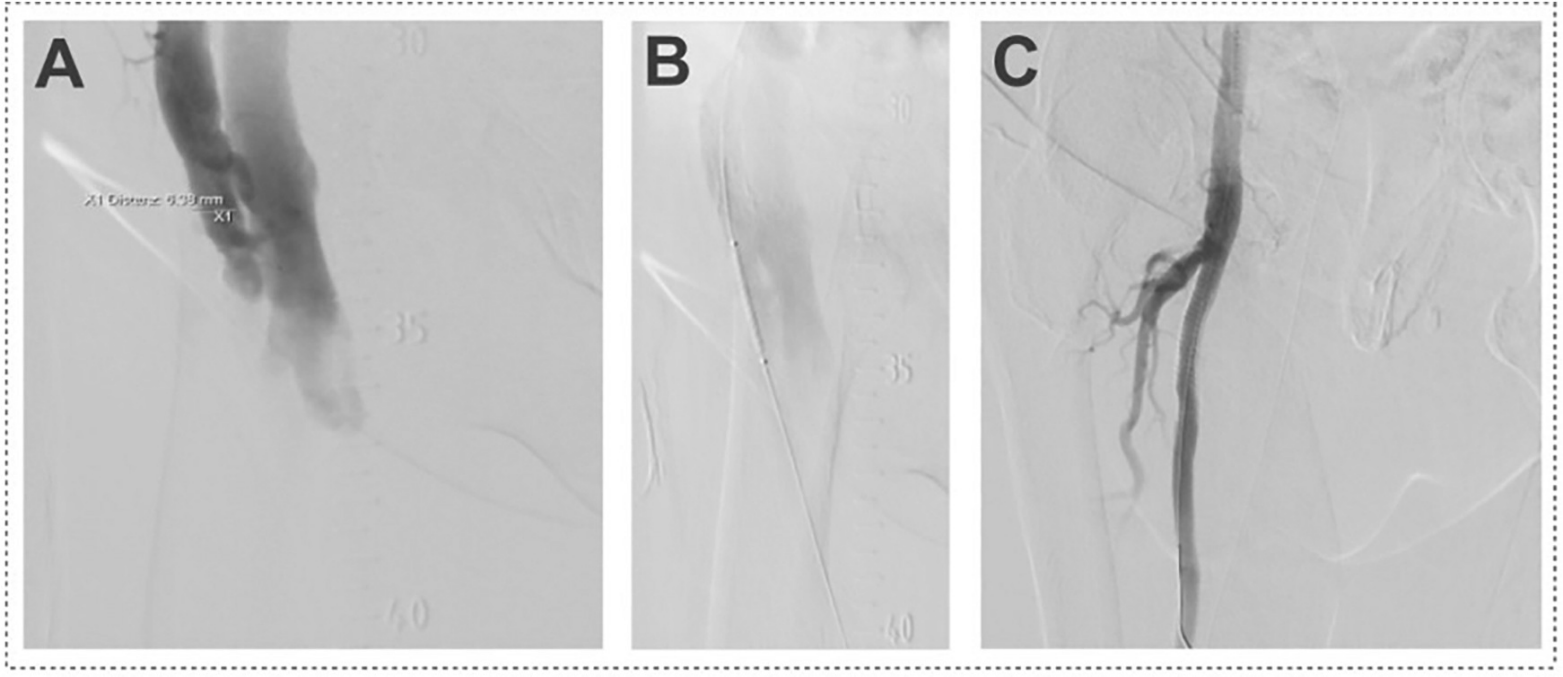
Iatrogenic arteriovenous (AV)-fistula after MitraClip procedure. Panel **(A)**: Cross over angiography shows AV-fistula between superficial femoral artery and common femoral vein. Panel **(B)**: Covered stent placed in the superficial femoral artery above AV-Fistula. Panel **(C)**: Angiography after implantation of covered stent in the superficial femoral artery showing no residual fistula.

There was a significant difference in unplanned surgical intervention between both groups. Surgical intervention was required in five patients in the Z-suture group (four patients due to AV-fistula with significant shunt volume, one patient due to pseudoaneurysm) whereas no surgical intervention was required in patients from the DPSS group (3.5% vs. 0%, *p* = 0.046, respectively). No significant difference was observed regarding requirement for endovascular intervention. Two patients in the Z-suture group received a covered stent in the femoral artery due to AV-Fistula (Figure 3). One patient in the DPSS group received a covered stent due to perforation of the right femoral vein (1.4% vs. 0.9%, *p* = 0.714 respectively). One patient from the Z-group required plastic surgery due to skin necrosis after delay to remove the Z-suture group. One patient from Z-suture group developed a postinterventional wound healing disorder. Skin necrosis and wound healing disorder were not documented in the DPSS group.

### Major adverse cardiac and cerebrovascular events (MACCE) and in-hospital mortality

MACCE was defined as myocardial infarction, stroke, and death. MACCE during hospitalization occurred in five patients in the Z-suture group and in five patients in the DPSS group (3.5% vs. 4.5%, *p* = 0.686, respectively). Two patients from the Z-suture group and three patients from DPSS group died during hospital stay (1.4% vs. 2.7%, *p* = 0.461, respectively).

## Discussion

Randomized controlled trials (RCTs) and real-world data demonstrated that PMVR is a generally safe and effective procedure (2, 4, 5, 7, 17), However, vascular and bleeding complications were described among the most frequent peri-interventional complications with incidences of about 10% to 20% depending on the definition and cohort (5, 18, 19), indicating the need for optimization of access site management. We therefore compared two different suture techniques to realize venous access closure and achieve haemostasis after removal of the 24-French sheath at the end of the MitraClip-procedure. Our study now reveals that large caliber venous access closure with subcutaneous absorbable DPSS technique was feasible, safe, and effective to achieve immediate haemostasis after MitraClip procedure. Compared with Z-suture, the use of DPSS closure was associated with a lower rate of postinterventional AV-fistula and required access related surgical intervention.

In the last decade, interventional therapies for valvular heart requiring large size sheath increased enormously. Notably, groin complications following cardiac interventions with a femoral approach are still a significant cause for morbidity, prolonged hospitalisation, and mortality (7, 20, 21). For arterial femoral access, a multitude of vascular occlusion devices have been examined and approved. In contrast, access site management for a venous femoral approach in procedures such as PMVR or electrophysiological intervention has been far less evaluated and only a limited variety of vascular occlusion devices and suture techniques to facilitate haemostasis and control vascular complications after using large size sheath have been investigated. Furthermore, vascular complications and definition of bleedings events are only infrequently reported in recent PMVR studies including large landmark RCTs (2, 4, 19, 22).

Several studies demonstrated the safety and efficacy of such Z-suture in patients after removing of venous sheath (10, 11, 23). Geis and colleagues compared the Z-suture technique (40 patients) with suture mediated occlusion device (Perclose ProGlide) for closure of femoral venous access (40 patients) after MitraClip-procedure (24). There was no difference between both groups regarding access related vascular complications. One patient in the ProGlide group had AV-fistula who required vascular surgery. Recently, Steppich and colleagues demonstrated in a retrospective study (277 patients) comparable results regarding VARC-2 minor and major access related vascular complications after application of the suture closure device ProGlide (127 patients) or the Z-suture technique (150 patients) (25). Of note, the vascular complication rates, incidence of AV-fistula, pseudoaneurysms and bleeding in our current study were in line with previous observational data examining the Z-suture technique for venous closure (5, 7, 14, 17). Occurrence of AV-fistula following a procedure via a venous access is unexpected at first glance, since arterial femoral puncture is not routinely performed during the MitraClip procedure. In some cases, AV-fistula might be the result of inadvertent arterial puncture. Principally, application of a large needle used for the Z-suture technique may result in accidental injury of the femoral artery. In contrast, the purse string suture technique is performed using a small needle and the DPSS technique may result in a more intensified and controlled pressure on the femoral vein access. Kypta and colleagues evaluated the use of subcutaneous absorbable DPSS after removal of large caliber venous sheaths to achieve immediate postprocedural haemostasis in 77 patients who underwent leadless pacemaker implantation via 23-French sheath and observed access site complications in three patients (3.9%) (14). More recently, Akkaya et al. showed in a retrospective study of 41 patients that DPSS technique was safe, effective, and associated with low rate of vascular complications after removal of 24-French sheath (26). Our observations in the DPSS group extend current knowledge from the above-mentioned studies.

Multivariable analysis of observational data suggested that transfusion of blood constitutes a predictor of in-hospital death following MitraClip procedure (7). Of note, less than a third of the bleedings requiring transfusion were related to the access site in our study. Körber et al. previously documented in a retrospective analysis of patients undergoing PMVR a similar ratio and revealed that obscure, but not access site related bleeding predicts peri-interventional acute kidney injury and associates with mortality and hospitalization (18). Remote bleeding was dominantly related with the puncture site of the central venous catheter as well as gastrointestinal and urethral bleeding.

Although the overall incidence of access site related vascular complication and bleeding events after MitraClip procedure was comparable in the Z-suture and DPSS group, the incidence of AV-fistula and unplanned surgical intervention was significantly lower after application of the DPSS technique. This finding and the numerically lower MVARC vascular complication rate may support a strategy of preferring the subcutaneous absorbable DPSS technique over Z-suture to achieve immediate haemostasis after MitralClip procedure. Furthermore, this technique provides a cosmetically attractive closure compared to Z-suture technique.

## Limitations

Our study has some limitations that should be considered when interpreting our results. The observational nature of our study and lack of randomization combined with the relatively small number of recruited patients and in particular the low event rate, introduces several limitations, including restricted generalizability, risk of random variability, lack of precision and reliability, and limited exploration of heterogeneity. Overall, the statistical power is limited in particular in regard to the analysis of subtypes of vascular complications and bleeding. Due to the non-randomized study design, baseline characteristics between the two groups differed in some respects such as the severity of MR with a greater EROA and Rvol, potentially affecting the rate of vascular complications. More than one clip was implanted in less patients of Z-group, which may result in a shorter duration of the procedure in the Z-suture group compared with the DPSS group. Furthermore, generalizability is restricted since we only provide data from a single center study and access site related complications are partially operator dependent. Although the operators involved in our study had abundant experience in venous access management, the sequential nature of the study design and an ongoing learning curve of the operators may impact our results. Prospective evaluation and further confirmatory analyses in larger, multi-center PMVR cohorts are needed. However, our analysis is the first study that compared two different subcutaneous suture techniques to achieve immediate haemostasis after MitraClip procedure and as an all-comers registry provides insights in real-world clinical practice.

## Conclusions

Subcutaneous absorbable DPSS technique was feasible, effective, and safe to achieve immediate haemostasis after MitraClip procedure with removal of large-caliber venous sheaths. Compared with Z-suture, the use of DPSS was associated with a lower rate of postinterventional AV-fistula, required access related surgical intervention and provided a cosmetically attractive closure.

## Data Availability

The raw data supporting the conclusions of this article will be made available by the authors, without undue reservation.
